# Accessory Gene Regulator (*agr*) Allelic Variants in Cognate *Staphylococcus aureus* Strain Display Similar Phenotypes

**DOI:** 10.3389/fmicb.2022.700894

**Published:** 2022-02-25

**Authors:** Li Tan, Yuyang Huang, Weilong Shang, Yi Yang, Huagang Peng, Zhen Hu, Yuting Wang, Yifan Rao, Qiwen Hu, Xiancai Rao, Xiaomei Hu, Ming Li, Kaisen Chen, Shu Li

**Affiliations:** ^1^College of Basic Medical Sciences, Army Medical University, Chongqing, China; ^2^Queen Mary College, Nanchang University, Nanchang, China; ^3^The First Affiliated Hospital of Nanchang University, Nanchang, China

**Keywords:** *Staphylococcus aureus*, quorum sensing system, accessory gene regulator (*agr*) alleles, *agr* polymorphisms, virulence factors

## Abstract

The accessory gene regulator (*agr*) quorum-sensing system is an important global regulatory system of *Staphylococcus aureus* and contributes to its pathogenicity. The *S. aureus agr* system is divided into four *agr* groups based on the amino acid polymorphisms of AgrB, AgrD, and AgrC. The *agr* activation is group-specific, resulting in variations in *agr* activity and pathogenicity among the four *agr* groups. Strains with divergent *agr* system always have different phenotypes. In the present report, we, respectively, exchanged the *agr* system of a certain *S. aureus* with other three *agr* alleles and assessed the corresponding phenotypes of these congenic strains. Replacement of the *agr* system led to significant variations in hemolytic activity, protein expression, and virulence gene expression comparing with that of the parental strain. Interestingly, we found that the biological characteristics of these *agr* congenic strains in the same strain background were highly similar to each other, and the allele-dependent differences of the *agr* systems were weakened. These findings indicate that the allele-dependent *agr* predilections of *S. aureus* are determined by some factors in addition to the polymorphisms of AgrB, AgrD, and AgrC. Future studies may reveal the novel mechanism to improve our understanding of the *agr* network.

## Introduction

The accessory gene regulator (*agr*) quorum-sensing system, a globe transcriptional regulator of *Staphylococcus aureus*, plays a key role in its pathogenesis and resistance (Bernabè et al., 2021) and has been intensively studied to aid drug and vaccine development (Tan et al., 2018). The *agr* locus comprises two adjacent transcripts, RNAII and RNAIII, which are controlled by P2 and P3 promoters, respectively (Ji et al., 1995). RNAII is composed of four genes, *agrB*, *agrD*, *agrC*, and *agrA* (Novick et al., 1995). *agrD* encodes the propeptide for an autoinducing peptide (AIP). *agrB* encodes an endopeptidase that is the processor of AIP. AgrC and AgrA, encoded by *agrC* and *agrA*, function as a two-component regulatory system. When *agr* system is activated, AIP propeptide is processed to an octapeptide by AgrB and secreted to extracellular space (Queck et al., 2008). As AIP reaches a threshold, the membrane-bound histidine kinase AgrC autophosphorylates and becomes activated, which leads to the phosphorylation of its cognate response regulator, AgrA (Queck et al., 2008). Phosphorylated AgrA activates the transcription of its own RNAII transcript to produce more AIP and also activates promoter P3 to increase the expression of RNAIII (Queck et al., 2008; Wang and Muir, 2016). The two main intracellular effectors, AgrA and RNAIII, regulate expression of virulence factors and contribute to the pathogenicity of *S. aureus* (Bronesky et al., 2016). AgrA can activate the synthesis of phenol soluble modulin (PSM) peptides, which are the only known toxins regulated by AgrA (Queck et al., 2008). RNAIII was shown to regulate primarily the expression of many important virulence factors and several transcriptional regulators (Wang and Muir, 2016).

The amino acid sequences of AgrB, AgrD, and AgrC are variable (Supplementary Figure 1), whereas AgrA, RNAIII, and their promoter regions are highly conserved (Supplementary Figure 2). According to the polymorphisms of AgrB, AgrD, and AgrC, the *agr* system in *S. aureus* is divided into four types named *agr*I, *agr*II, *agr*III, and *agr*IV. Each *agr* variant produces its own specific AIP, which triggers autoinduction. The heterologous pairing of AIP inhibits the response of other *agr* types and leads to heterologous mutual inhibition (Ji et al., 1997). The variations of *agr* specificity may form specific functional units that drive evolutionary diversification in *Staphylococcus* and also have significant implications for host disease (Wright et al., 2005). Cues between special *agr* groups and some disease predilections have been reported by previous studies. For example, most of *agr*II clinical *S. aureus* strains are isolated from acute infection, and half of clinical methicillin-resistant *S. aureus* (MRSA) bloodstream isolates are in the *agr*II group. Menstrual toxic shock syndrome is usually caused by *agr*III and *agr*IV *S. aureus* (Jarraud et al., 2002), and *agr*IV strains are associated with exfoliative syndromes and bullous impetigo (Gomes et al., 2005). The *agr*II and *agr*IV strains exhibit higher biofilm formation capacity (Khoramrooz et al., 2016). The distributions of certain toxin genes and mobile genetic elements also show *agr* group specificity (Wright et al., 2005). It has been reported that the specific lineage and geographical distribution of *S. aureus* may correlate with *agr* types (Holtfreter et al., 2007). In summary, clinical symptoms caused by some staphylococcal strains are closely associated with their *agr* subgroups (Traber et al., 2008). However, it is not known whether there are any other factors involved in the *agr* predilection and *agr-s*pecific virulence genotypes of *S. aureus* in addition to the specific *agr* type, or the polymorphous AgrB, AgrD, and AgrC.

In this study, we replaced the *agr* system of *S. aureus* Newman strain (*agr*I type) or N315 (*agr*II type) with different *agr* alleles. The resulting four congenic strains were assessed for their *agrA* activity, hemolytic activity, pigmentation, exoprotein expression, and virulence factor expressions.

## Materials and Methods

### Bacterial Strains, Plasmids, Primers, and Growth Conditions

*S. aureus* strain Newman, an *agr* group I prototype used as the backbone strain in this study, was originally isolated from a secondarily infected tubercular osteomyelitis lesion (Duthie and Lorenz, 1952). N315, an *agr*II prototype MRSA with defective *agr* system, was also used as control background strain (Tsompanidou et al., 2011). MW2 is a prototypical *agr* group III strain originally isolated from a child with fatal septicemia and septic arthritis (Mei et al., 2011). XQ is a clinical community-associated MRSA belongs to group IV prototype strain isolated from an adolescent patient with staphylococcal scalded skin syndrome (Rao et al., 2015). *Escherichia coli* strain DH5α was used for plasmid construction and genetic manipulation. All strains were stored in 10% glycerol at −80°C (Supplementary Table 1). *E. coli* strains were grown in Luria–Bertani broth, whereas *S. aureus* strains were cultivated in trypticase soy broth (TSB; Sigma) or brain–heart infusion medium (Sigma). When necessary, ampicillin (100 μg/mL) and chloramphenicol (20 μg/mL) were added to the medium. The temperature-sensitive *S. aureus* plasmid pBT2 and *E. coli*–*S. aureus* shuttle plasmid pLI50 kindly provided by Prof. Baolin Sun (University of Science and Technology of China, China) were used for gene mutation and complementation assay, respectively. Transformations of *S. aureus* strains were performed by electroporation (Bio-Rad Gene Pulser). The primers used in this study were presented in Supplementary Table 2.

### Construction of Accessory Gene Regulator Gene Markerless Deletion Mutant

The NewmanΔ*agrBDC* and N315Δ*agrBDCA* markerless deletion mutants were constructed with pBT2 plasmid using homologous recombinant strategy as described previously (Yuan et al., 2013). Take NewmanΔ*agrBDC* construction as an example, a 803-bp DNA fragment upstream of *agrBDC* locus was amplified from Newman genome DNA using primer pairs, Δ*agrBDC* up-for and Δ*agrBDC* up-rev (Supplementary Table 2). The polymerase chain reaction (PCR) product was digested with *Hin*dIII and *Sal*I and subcloned into the same site of pBT2 to obtain plasmid p*agrBDC*F. Then, a 957-bp DNA fragment downstream of *agrBDC* locus was amplified and subcloned into the *Bam*HI and *Eco*RI site of p*agrBDC*F, yielding the Δ*agrBDC* knockout plasmid (p*agrBDC*). The p*agrBDC* plasmid was identified by restriction enzyme digestion and DNA sequencing and then electrotransformed into *S. aureus* RN4220 and Newman to construct NewmanΔ*agrBDC* markerless deletion mutant by homologous recombination. The mutants were screened and confirmed by PCR amplification and DNA sequencing. The N315Δ*agrBDCA* markerless deletion mutant was constructed with similar strategy.

### Construction of Accessory Gene Regulator Gene Allele Replacement Strains

The *agrBDC* fragments of *agr*II, *agr*III, and *agr*IV were separately obtained from N315, MW2, and XQ by PCR amplification and were, respectively, inserted into the *Sal*I and *Bam*HI sites of pBT2 plasmid to yield p*agrBDC*-II, p*agrBDC*-III, and p*agrBDC*-IV knock-in vectors. Then, these p*agrBDC* vectors were sequentially introduced into *S. aureus* RN4220 and NewmanΔ*agrBDC* deletion mutant strain to construct allelic replacement strains *via* homologous recombination. The substitution of *agrBDC* fragments of allelic congenic strains was also verified by PCR amplification and DNA sequencing.

### Construction of *agrBDCA* Plasmid Complemented Strains

Full gene DNA of types I through IV *agr* clusters and their promoters were separately amplified from Newman, N315, MW2, and XQ using the primers described in Supplementary Table 2 and subcloned into the pLI50 plasmid. All positive recombinant plasmids were confirmed by DNA sequencing. Then, these plasmids were, respectively, transformed into *E. coli* DH5α, *S. aureus* RN4220, and finally into NewmanΔ*agrBDC* or N315Δ*agrBDCA* mutant to construct types I–IV *agrBDCA* genes complemented strains.

### Biological Characteristic Analysis

The hemolytic activity, pigment formation, exoprotein production, and gene transcription of *agr* allelic congenic strains were analyzed to assess the influence of *agr* system replacement on *S. aureus* biological characteristics.

For hemolytic activity assessment, overnight culture of each single *S. aureus* colony was diluted into the same colony-forming units (CFUs) and plated on rabbit’s blood agar plates, followed by overnight growth at 37°C for hemolysis analysis. Furthermore, the hemolytic activity was also evaluated according to the hemolysis of rabbit erythrocytes. As the method described previously (Pader et al., 2014), 100 μL overnight culture supernatant was mixed with 6% rabbit blood in phosphate-buffered saline and then incubated at 37°C for 20 min. The unlysed blood cells were removed by centrifugation, and the erythrocyte lysis was determined with the OD543 values of supernatant.

To evaluate staphyloxanthin production, the diluted overnight inoculation of *S. aureus* strains was also cultured on TSB plate to assess the effect of *agr* replacement on pigment formation. Moreover, staphyloxanthin productions were also quantitatively analyzed and adapted from a previously published method (Liu et al., 2018). Cells in 1-mL overnight culture were collected and washed thrice with sterilized water and then resuspended with 200 μL methanol and heated at 55°C for 3 min. After the cells were centrifuged at 10,000 × *g* for 1 min, the OD462 value of supernatant was detected.

The overnight culture supernatants of *S. aureus* were collected by centrifugation. Proteins in supernatants were precipitated with trichloroacetic acid and analyzed by sodium dodecyl sulfate–polyacrylamide gel electrophoresis (SDS-PAGE) in a 12% polyacrylamide gel.

The change in gene transcription was appraised with quantitative real-time PCR (qRT-PCR). Cells were harvested at 6 h after inoculation, which represent the mid-log phase of the *S. aureus* growth. The total bacterial RNA was isolated using SV Total RNA Isolation System kit (Z3100; Promega, United States). The contaminated genomic DNA in RNA was degraded with DNaseI. The cDNA was prepared using PrimeScript RT Reagent Kit (RR047A; Takara, Japan) and used for qRT-PCR using GoTaq ^®^ qPCR Master Mix (A6001; Promega, United States) on an ABI SimpliAmp PCR detection system (United States). Specific primers for qRT-PCR (Supplementary Table 3) were designed according to the target gene sequences. All PCR reactions were performed in triplicate, with 16S rDNA as internal control. The relative expression of gene products was normalized to the housekeeping gene 16S and calculated using the 2^–ΔΔCT^ method.

## Results

### Construction of Congenic Strains Containing Accessory Gene Regulator Alleles

**FIGURE 1:**
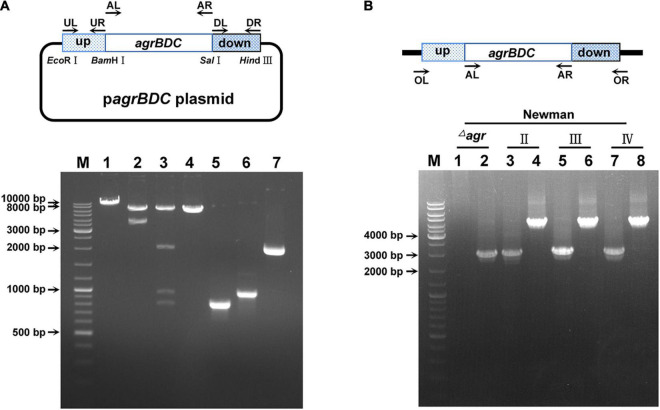
The identification of *agr* allelic mutant in *S. aureus* Newman. **(A)** The confirmation of *agr* allele replacement plasmid by restriction enzyme digestion (take type II *agrBDC* replacement as an example). Bands 1, 2, and 3 are the single (*Bam*HI), double (*Eco*RI + *Hin*dIII), and four (*Bam*HI, *Eco*RI, *Hin*dIII, and *Sal*I) enzymes digestion patterns of *agrBDC*-II allelic replacement plasmid, respectively. Band 4 is the pattern of empty pBT2 plasmid linearized by *Bam*HI. Bands 5, 6, and 7 are patterns of the PCR amplification products of *agrBDC*-up (Band5, primers UL + UR), *agrBDC*-down (Band6, primers DL + DR), and *agrBDC*-II from N315 (Band7, primers AL + AR) fragments, respectively. **(B)** The confirmation of *agr* allelic replacement strains in Newman background by PCR amplification with primers AL + AR (Bands 1, 3, 5, 7) and primers OL + OR (Bands 2, 4, 6, 8). Bands 1–2 refer to the PCR products of NewmanΔ*agrBDC* mutants, Bands 3–4 are the PCR products of the congenic strain harbor *agrBDC*-II sequence, bands 5–6 refer to PCR products of the congenic strain with *agrBDC*-III replacement, and bands 7–8 for *agrBDC*-IV replacement. All primers are shown in Supplementary Table 2.

The *agr* systems were divided into four *agr* groups named *agr*I, *agr*II, *agr*III, and *agr*IV in *S. aureus* (Wang and Muir, 2016). AgrA is highly conserved, whereas AgrB, AgrD, and AgrC are variable among the four *agr* groups (Supplementary Figure 1). In this study, *S. aureus* Newman and N315 were selected for *agr* congenic strains construction to assess the effects of divergent *agr* alleles. The whole RNAII transcripts (involved *agrB*, *agrD*, *agrC*, and *agrA* genes) were allelic substituted to construct congenic strains in N315, whereas only *agrB*, *agrD*, and *agrC* genes were replaced in Newman congenic strains. The congenic strains were constructed *via* homologous recombination and confirmed by PCR amplification (Figure 1 and Supplementary Table 2) and DNA sequencing.

### Effects of Accessory Gene Regulator Alleles on Hemolytic Activity

**FIGURE 2:**
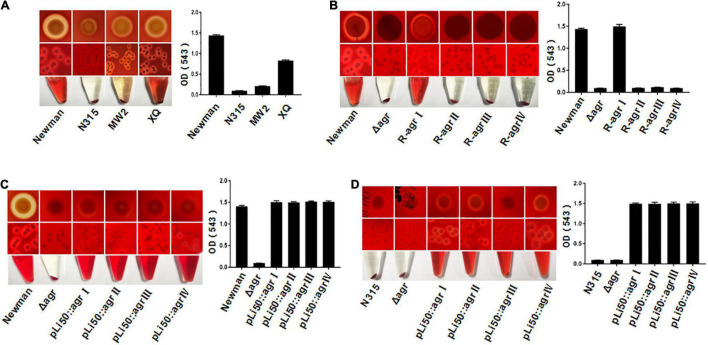
Effects of *agr* alleles on the hemolytic activities of *S. aureus*. The overnight cultures of *S. aureus* strains with the same CFUs were plated on Columbia blood agar plates and grown for 24 h at 37°C. **(A)** Hemolytic activities of the four wild *agr* allelic strains: *agr*I (Newman), *agr*II (N315), *agr*III (MW2), and *agr*IV (XQ). **(B)** Hemolytic activities of the wild Newman strain, NewmanΔ *agrBDC*, and *agr* genomic *in situ* replacement mutants of Newman (*agr*I to *agr* IV). Hemolytic activities of the wild type, Δ*agrBDC* mutants and pLI50-*agr* plasmid complement mutants of Newman **(C)** and N315 **(D)**. The hemolytic activities of the relative strains were determined by measuring the optical density (OD543).

*S. aureus* is able to secrete a variety of toxins, such as α-hemolysin, bicomponent leukocidins, γ-hemolysin, Panton–Valentine leukocidin (PVL), β-hemolysin, δ-hemolysin, PSMs, and so on (Cheung and Otto, 2012). These exotoxins have leukotoxic and hemolytic activities and are widely associated with the pathogenicity of *S. aureus* (Powers et al., 2015; Seilie and Bubeck Wardenburg, 2017). α-Hemolysin, γ-hemolysin, PVL, δ-hemolysin, and PSM belong to pore-forming toxins capable of forming transmembrane aqueous channel and leading to host cell lysis (Reyes-Robles and Torres, 2017). β-Hemolysin is a neutral sphingomyelinase capable of digesting sphingomyelin into ceramide and phosphorylcholine (Vandenesch et al., 2012). The production of hemolysins in *S. aureus* is tightly regulated, and the *agr* system plays an important role in this process (Johansson et al., 2019). It was reported that the *agr* system controls the expression of α-, β-, and γ-hemolysin and PVL by RNAIII and regulates the transcription of δ-hemolysin and PSMs through AgrA (Arya and Princy, 2013; Singh and Ray, 2014). The hemolytic capabilities of *S. aureus* vary with their *agr* types. As shown in Figure 2A, the Newman (*agr*I) and XQ (*agr*IV) strains have strong hemolytic activities, the MW2 (*agr*III) strain showed weak hemolysis, and the N315 (*agr*II) strain presented the lowest hemolytic toxicity (Figure 2A).

To assess the effects of different *agr* alleles on *S. aureus* hemolytic activity, the *agrBDC* genes in Newman strain (*agrBDC*-I) were deleted and *in situ* substituted with other three *agrBDC* alleles (*agrBDC*-II, *agrBDC*-III, and *agrBDC*-IV). As expected, the hemolytic activity of the NewmanΔ*agrBDC* strain was significantly lower than that of the wild strain (Figure 2B). The knock-in of type I *agrBDC* genes back to the genome of NewmanΔ*agrBDC* mutant recovered the hemolytic activity of revertant strain (Figure 2B). Contrary to our expectations, no visible hemolysis and very low hemolytic activity were discovered on three congenic replacement strains harboring heterologous *agr* systems (Figure 2B). The hemolytic activity controlled by *agr* system seemed to be severely suppressed when the three heterologous *agr* alleles were *in situ* recombined into the genome of NewmanΔ*agrBDC* mutant.

To investigate this further, four recombinant pLI50 plasmids containing types I to IV *agrBDCA* genes and their promoter sequences were, respectively, transformed into the Δ*agrBDC* mutant strains of Newman or N315. As shown in Figures 2C,D, the four *agr* complemented strains showed similar hemolytic activities to each other. The differences in hemolysis across *agr* groups were significantly weakened in the same Newman or N315 background when compared with the standard *agr* allelic strains.

### Effects of Accessory Gene Regulator Alleles on Pigment Formation

**FIGURE 3:**
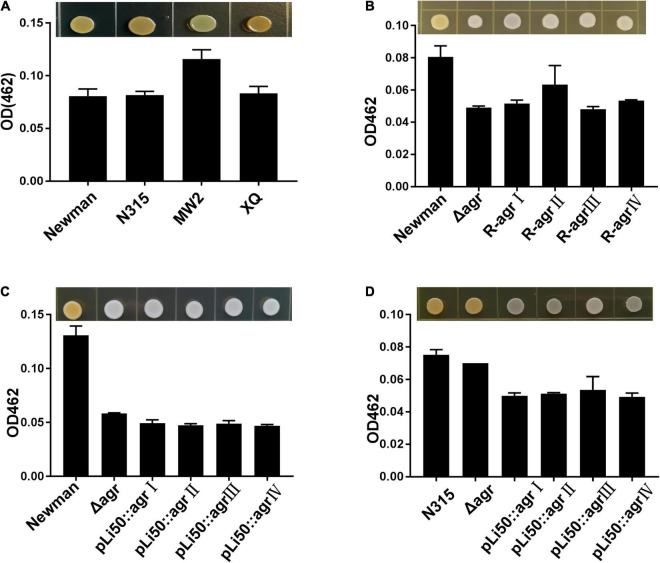
Effects of *agr* alleles on the pigment formations of *S. aureus*. The overnight cultures of *S. aureus* strains with the same CFUs were plated on TSB agar plates and grown for 24 h at 37°C. **(A)** Pigment productions of the four wild *agr* allelic strains: *agr*I (Newman), *agr*II (N315), *agr*III (MW2), and *agr*IV (XQ). **(B)** Pigment productions of the wild Newman strain, NewmanΔ*agrBDC*, and *agr* genomic *in situ* replacement mutants (R-*agr*Ito *agr*IV) of Newman. Pigment productions of the wild type, Δ*agrBDC* mutants, and pLI50-*agr* plasmid complement mutants of Newman **(C)** and N315 **(D)**. Pigment productions of the relative strains were determined by measuring the optical density (OD462).

Staphyloxanthin, a carotenoid pigment produced by *S. aureus*, protects bacteria from neutrophil oxidants (Xue et al., 2019). When the *agrBDC* sequence was deleted, NewmanΔ*agrBDC* mutant strain lost staphyloxanthin production and formed white colonies (Figures 3B,C). All congenic strains of Newman constructed by genomic replacement or plasmid complemented did not bring back the yellow color of wild Newman strain and showed white colonies (Figures 3B,C). The deletion of genes *agrBDCA* of N315 did not lead to visible color change (Figure 3D). However, four *agr* alleles complemented mutants in N315 presented white colonies in contrast to the golden colonies from the wild N315 and N315Δ*agrBDCA* mutant strains (Figure 3D).

**FIGURE 4:**
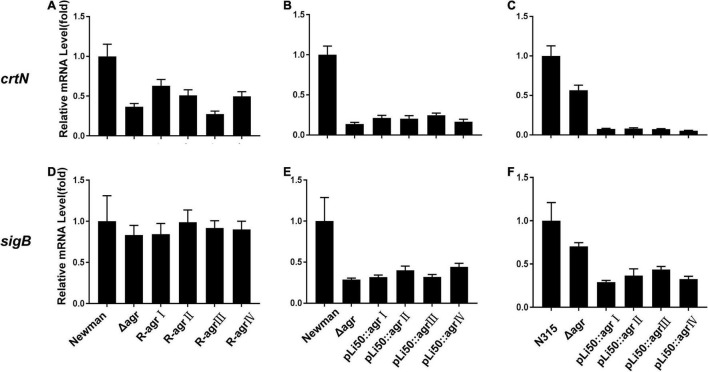
Effects of *agr* alleles on the transcriptions of staphyloxanthin biosynthesis–related genes. The relative transcription levels of *crtN* **(A–C)** and *sigB* **(D–F)** were determined. **(A,D)** The relative transcription levels of wild Newman strain, NewmanΔ*agrBDC* mutant, and *agr* genomic *in situ* replacement mutants (R-*agr*Ito *agr*IV) of Newman. **(B,E)** The relative transcription levels of wild Newman strain, NewmanΔ*agrBDC* mutant, and four *agr* plasmid complemented strains in Newman background. **(C,F)** The relative transcription levels of wild N315 strain, N315Δ*agrBDCA* mutant, and four *agr* plasmid complemented strains in N315 background.

Five genes, *crtOPQMN*, organized in an operon are responsible for the biosynthesis of staphyloxanthin (Xue et al., 2019). Two colorless farnesyl diphosphate successively catalyzed by five enzymes (CrtM, CrtN, CrtP, CrtQ, CrtO) to yield orange staphyloxanthin. Dehydrosqualene desaturase, CrtN, catalyzes the formation of the first deep yellow-colored carotenoid intermediate product, 4,4′-diaponeurosporene (Wieland et al., 1994). The sigma factor B (SigB) plays an essential role in regulating staphyloxanthin biosynthesis by binding to the promoter that laid in upstream of *crtO* (Kullik et al., 1998; Pelz et al., 2005). In the present study, the transcription levels of genes *crtN* and *sigB* were analyzed and found to be down-regulated in congenic strains when compared with the wild strains, Newman or N315 (Figure 4). It can be inferred that the down-regulated transcriptions of *crt* operon, especially that of *crtN*, are responsible for the weaken pigmentations of congenic strains, and the decreased SigB expression may play an important role in these processes.

### Effects of Accessory Gene Regulator Alleles on Exoprotein Productions

**FIGURE 5:**
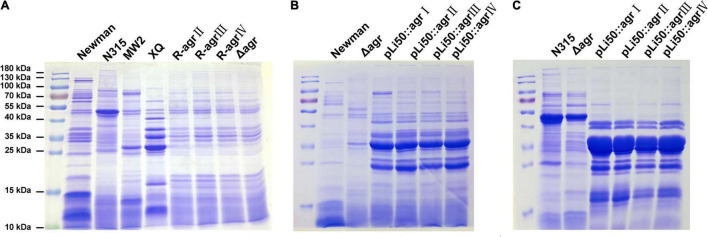
Effects of *agr* alleles on the exoprotein productions of *S. aureus*. Overnight culture supernatants of *S. aureus* strains were collected and analyzed with SDS-PAGE. **(A)** Exoprotein profiles of the four wild *agr* allelic strains (*agr*I/Newman, *agr*II/N315, *agr*III/MW2, and *agr*IV/XQ), NewmanΔ*agrBDC* mutant and three *agr* genomic *in situ* replacement strains in Newman background. **(B)** Exoprotein profiles of wild Newman strain, NewmanΔ*agrBDC* mutant, and four *agr* plasmid complemented strains in Newman background. **(C)** Exoprotein profiles of wild N315 strain, Δ*agrBDCA*-N315 mutant, and four *agr* plasmid complemented strains in N315 background.

To explore the influences of *agr* alleles on *S. aureus* exoprotein expressions, overnight culture supernatants were collected and analyzed by SDS-PAGE. As shown in Figure 5A, each *agr* type strain has its distinctive exoprotein profiles. Deletion of *agrBDC* genes led to significant changes in exoprotein expressions of Newman (Figure 5A). The knock-in of *agr*II, *agr*III, or *agr*IV *agrBDC* genes did not change the exoprotein expressions of NewmanΔ*agrBDC*; the three allelic strains exhibited similar exoprotein profiles as that of NewmanΔ*agrBDC* mutant (Figure 5A). However, when the four allelic *agrBDCA* genes were introduced by the pLI50 plasmid, the exoprotein patterns of four *agr* allelic complemented strains changed, which were highly similar to each other (Figure 5B) but different from those of wild *agr* allelic strains and the genomic replacement strains (Figures 5A,B). The *agr* allele–dependent difference in exoprotein expression was diminished when in the same Newman background. The similar results were also observed in N315 (Figure 5C). These complement strains exhibited significantly higher exoprotein expression than wild strains (Figures 5B,C). The two most abundant bands at approximately 25 and 35 kDa were identified as bicomponent hemolysin, α-hemolysin, or serine protease with mass spectrometry (Supplementary Tables 4, 5), which have been demonstrated to be regulated by *agr* system.

### Effects of Accessory Gene Regulator Alleles on Virulence Gene Regulations

**FIGURE 6:**
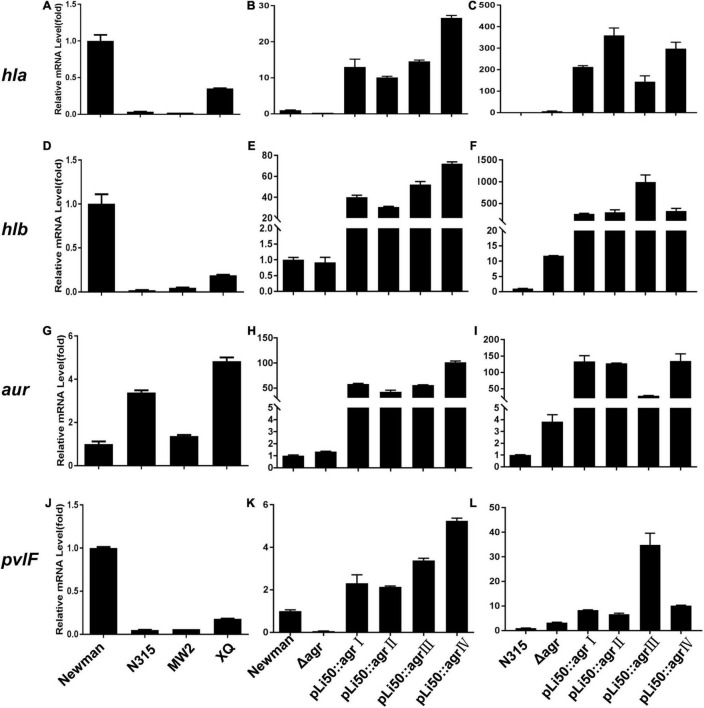
Effects of *agr* alleles on virulence gene transcriptions of *S. aureus.* The relative transcription levels of *hla* **(A–C)**, *hlb* **(D–F)**, *aur* **(G–I)**, and *pvlF* **(J–L)** among the four wild *agr* allelic strains (*agr*I/Newman, *agr*II/N315, *agr*III/MW2, and *agr*IV/XQ) (left); *agr* plasmid complemented congenic strains in Newman (middle); or N315 (right) background were analyzed. Error bars represent the average standard deviation (SD) of three separate experiments.

The ability of *S. aureus* to invade host tissues and cause infections depends on the production of a number of virulence factors. Many of them are regulated by the *agr* system such as α-toxin, β-toxin, δ-toxin, serine protease, fibrinolysin, PSM, and enterotoxin B (Seilie and Bubeck Wardenburg, 2017). In this study, we sought to examine the effects of different *agr* alleles on the transcription of virulence factors as *hla*, *hlb*, *hld*, *hlg*, *psm*α, *psm*β, *pvl*, and *aur* (Figure 6 and Supplementary Figure 4).

We found that the transcription of most toxins that are known to be activated by *agr* system (Bronesky et al., 2016) was now down-regulated in the NewmanΔ*agrBDC* mutant strain; the transcriptional factors and some surface proteins (Foster et al., 2014) that are usually inhibited by *agr* were now up-regulated. When the *agrBDC* genes of Newman strain (*agr*I) were genomically replaced by *agrBDC* genes from the other three *agr* alleles, the resulting mutants showed very low *agr* expression (data not shown). This is consistent with the finding of hemolytic assay, staphyloxanthin formation test, and exoprotein profile analysis. As the *agrBDC* deletion was complemented by plasmid pLI50, the transcription of *agrA* and RNAIII of NewmanΔ*agrBDC* returned to the original level of wild Newman (Supplementary Figures 3B,E). While the relative mRNA level of *agrA* increased 30–50 times and the RNAIII increased about thousand folds compared with that of wild N315 (Supplementary Figures 3C,F), the virulence factor transcription of these allelic complemented strains constructed in the same background varied in the same trend, showing an elimination of *agr*-dependent difference in identical background (Figure 6 and Supplementary Figure 4). For example, the transcription levels of *hla* gene of four *agr* congenic Newman strains increased 15–30 times compared with the wild Newman (Figure 6B), whereas they significantly increased 150–300 times when the four *agr* alleles were, respectively, complemented in *agr* defective N315 by plasmid pLI50 (Figure 6C). The *hlb* mRNA level showed 260- to 990-fold increase in N315 background, whereas only 30- to 72-fold in Newman background (Figures 6E,F). Similar changes were also observed in transcriptions of proteases such as aureolysin-encoding gene (*aur*, Figures 6H,I) and PVL-encoding gene (*pvl*, Figures 6K,L). Interestingly, the levels of *psm*α and *psm*β in all the four complemented strains were significantly decreased in the Newman background (Supplementary Figures 4H,K) compared with the wild Newman strain, but increased hundreds or thousands of folds in the N315 background (Supplementary Figures 4I,L).

## Discussion

The *agr* quorum-sensing system is a global regulator in *S. aureus* and controls the expression of numerous surface molecules, secreted enzymes, and cytotoxins (Ford et al., 2020). The amino acid polymorphisms in AgrB, AgrD, and AgrC separate the *agr* system into four groups. Strains with divergent *agr* groups also show different biological phenotypes and virulence factor production. However, the influence factors of the *S. aureus agr-s*pecific genotypes and virulence remain poorly understood. To dissect the contributions of four divergent *agr* alleles on *S. aureus* biological properties, Geisinger et al. (2012) constructed *agr* congenic strains by individually inserting different *agr* allele at the staphylococcal pathogenicity island (SaPI)-1 *attC* locus site of the same background strain. They found that divergent *agr* alleles showed different *agr* activation dynamic and virulence factor production, and the allele-dependent differences are mediated by the polymorphisms in *agrBDCA* genes (Geisinger et al., 2012). In our study, the *agrBDC* genes of Newman (*agr*I) and the *agrBDCA* genes of N315 (*agr*II) were, respectively, replaced with those of other three *agr* groups by *in situ* genomic substitution or plasmid complementation. As expected, the knockout of *agrBDC* genes led to significant variations in hemolysis activity, pigment formation, exoprotein expression, and virulence factor expressions of NewmanΔ*agrBDC* mutant. No discernible difference was observed between wild N315 and N315Δ*agrBDCA* mutant because of the low activity of the N315 *agr* system under normal condition (Figures 2–6). However, when the three heterologous *agr* alleles (*agr*II, *agr*III, and *agr*IV) were individually introduced into the native *agr* site of NewmanΔ*agrBDC* genome, the hemolytic activity, pigment formation, and exoprotein expression of three congenic strains were almost identical to those of NewmanΔ*agrBDC* mutant. In addition, the *agrBDCA* and promoter regions of four *agr* alleles were also introduced into *S. aureus* Δ*agrBDC* strain (Newman or N315) by plasmid complementation. The hemolysis, exoprotein expression, pigment formation and virulence gene transcription of these plasmid complemented congenic strains were different from the wild strains harboring divergent *agr* allele (Newman for *agr*I, N315 for *agr*II, MW2 for *agr*III, and XQ for *agr*IV) and background strains (Newman or N315), but exactly similar to each other. According to the findings of our study, the congenic strains present similar biological properties when different *agr* alleles are individually introduced in the same background strain whether by *in situ* genomic replacement or plasmid complement. It appears that the *agr* allele–dependent differences were weakened when in an identical background, which is distinct to the study by Geisinger et al. (2012) Perhaps, there are some other factors involved in the presentation of *agr*-controlled biological phenotype in addition to the polymorphous in *agrBDCA* genes.

The *agr* system is a critical regulatory system in *S. aureus.* Four allelic variants were reported, and each *agr* variant mediates the autoinduction of its own AIP (Jarraud et al., 2000). The *agr* cross-inhibition may drive evolutionary diversification in *S. aureus*. Our results indicate that cross-inhibition driven by *agr* polymorphisms may be affected by additional unknown factors in addition to the polymorphous of AgrBDCA proteins. It is well known that the *S. aureus agr* system can regulate the transcriptions of many genes and also be directly and indirectly controlled by other regulators (Queck et al., 2008). For example, SarA (Arya and Princy, 2013), SarU (Manna and Cheung, 2003), and MgrA (Manna and Cheung, 2006) are reported to up-regulate the expression of the *agr* system, whereas SigB (Bischoff et al., 2001), SarX (Manna and Cheung, 2006), and CodY (Majerczyk et al., 2010) may lead to the down-regulation of the *agr* system. The transcription levels of *sarA* and *sigB* were analyzed in this study (data not shown). Unfortunately, no direct correlation was observed between the expressions of these regulators and the similar phenotypes of *agr* allelic congenic strains. More in-depth studies are needed to reveal the putative factors and regulation mechanism, which may improve our understanding of the *S. aureus agr* system.

Taken together, our results further indicate that diversities of AgrB, AgrD, and AgrC contribute to the allele-dependent differences in *agr-r*egulated activities in *S. aureus*. Additional unknown factors may also interfere with the *agr-r*egulated phenotypes in *S. aureus*.

## Data Availability Statement

The raw data supporting the conclusions of this article will be made available by the authors, without undue reservation.

## Author Contributions

SL, XH, and XR conceived and designed this study. LT, SL, and YH carried out the experiments and analyzed the results. YW, ZH, and YR gave important suggestions to the data interpretation. WS, YY, and HP provided guidance in performing the experiments. XR, QH, ML, and KC provided guidance on the ideas for the study. All authors discussed the results and commented on the manuscript.

## Conflict of Interest

The authors declare that the research was conducted in the absence of any commercial or financial relationships that could be construed as a potential conflict of interest.

## Publisher’s Note

All claims expressed in this article are solely those of the authors and do not necessarily represent those of their affiliated organizations, or those of the publisher, the editors and the reviewers. Any product that may be evaluated in this article, or claim that may be made by its manufacturer, is not guaranteed or endorsed by the publisher.

## Supplementary Material

Supplementary Figure 1 The amino acids comparisons of AgrB **(A)**, AgrD **(B)**, and AgrC **(C)** among the four *S. aureus agr* allelic strains: *agr*I (Newman), *agr*II (N315), *agr*III (MW2), and *agr*IV (XQ). The sequences were obtained from NCBI (National Center for Biotechnology Information) database and aligned with the Clustal X and DNAMAN software. The blast results show that the amino acid sequences of AgrB, AgrD, and AgrC are variable.

Supplementary Figure 2 The comparisons of AgrA **(A)**, RNAIII **(B)**, and promoter sequences **(C)** among the four *S. aureus agr* allelic strains: *agr*I (Newman), *agr*II (N315), *agr*III (MW2), and *agr*IV (XQ). The sequences were obtained from NCBI (National Center for Biotechnology Information) database (http://www.ncbi.nlm.nih.gov) and aligned with the Clustal X and DNAMAN software. The blast results show that the AgrA, RNAIII, and promoter region are highly conserved among the four *S. aureus agr* allelic strains.

Supplementary Figure 3 Effects of *agr* alleles on *agr* transcriptions. The total bacterial RNA was isolated in mid-log phase, and gene transcription was appraised with quantitative RT-PCR. The transcription of *agrA* **(A–C)** and RNAIII **(D–F)** among the four wild *agr* allele strains (*agr*I/Newman, *agr*II/N315, *agr*III/MW2, and *agr*IV/XQ) (left) and *agr* plasmid complemented congenic strains in Newman (middle) or N315 (right) background were analyzed. Error bars represent the average standard deviation (SD) of three separate experiments.

Supplementary Figure 4 Effects of *agr* alleles on virulence gene transcriptions of *S. aureus*. The total bacterial RNA was isolated in mid-log phase, and gene transcription was appraised with quantitative RT-PCR. The transcription of *hlgB* **(A–C)**, *hld* **(D–F)**, *psm*α **(G–I)**, and *psm*β **(J–L)** among the four wild *agr* allelic strains (*agr*I/Newman, *agr*II/N315, *agr*III/MW2, and *agr*IV/XQ) (left); *agr* plasmid complemented congenic strains in Newman (middle); or N315 (right) background were analyzed. Error bars represent the average standard deviation (SD) of three separate experiments.

## References

[B1] Arya R. Princy S. (2013). An insight into pleiotropic regulators Agr and Sar: molecular probes paving the new way for antivirulent therapy. *Future Microbiol.* 8 1339–1353. 10.2217/fmb.13.92 24059923

[B2] Bernabè G. Dal Pra M. Ronca V. Pauletto A. Marzaro G. Saluzzo F. (2021). A novel Aza-derivative inhibits quorum sensing signaling and synergizes Methicillin-Resistant *Staphylococcus aureus* to clindamycin. *Front. Microbiol.* 12:610859. 10.3389/fmicb.2021.610859 33633702 PMC7899991

[B3] Bischoff M. Entenza J. M. Giachino P. (2001). Influence of a functional sigB operon on the global regulators sar and agr in *Staphylococcus aureus*. *J. Bacteriol.* 183 5171–5179. 10.1128/JB.183.17.5171-5179.2001 11489871 PMC95394

[B4] Bronesky D. Wu Z. Marzi S. Walter P. Geissmann T. Moreau K. (2016). *Staphylococcus aureus* RNAIII and its regulon link quorum sensing, stress responses, metabolic adaptation, and regulation of virulence gene expression. *Ann. Rev. Microbiol.* 70 299–316. 10.1146/annurev-micro-102215-095708 27482744

[B5] Cheung G. Otto M. (2012). The potential use of toxin antibodies as a strategy for controlling acute *Staphylococcus aureus* infections. *Expert Opin. Ther. Targets* 16 601–612. 10.1517/14728222.2012.682573 22530584 PMC3354029

[B6] Duthie E. Lorenz L. (1952). Staphylococcal coagulase; mode of action and antigenicity. *J. Gen. Microbiol.* 6 95–107. 10.1099/00221287-6-1-2-95 14927856

[B7] Ford C. Hurford I. Cassat J. (2020). *Staphylococcus aureus* antivirulence strategies for the treatment of infections: a mini review. *Front. Microbiol.* 11:632706. 10.3389/fmicb.2020.632706 33519793 PMC7840885

[B8] Foster T. Geoghegan J. Ganesh V. Höök M. (2014). Adhesion, invasion and evasion: the many functions of the surface proteins of *Staphylococcus aureus*. *Nat. Rev. Microbiol.* 12 49–62. 10.1038/nrmicro3161 24336184 PMC5708296

[B9] Geisinger E. Chen J. Novick R. (2012). Allele-dependent differences in quorum-sensing dynamics result in variant expression of virulence genes in *Staphylococcus aureus*. *J. Bacteriol.* 194 2854–2864. 10.1128/jb.06685-11 22467783 PMC3370610

[B10] Gomes A. Vinga S. Zavolan M. de Lencastre H. (2005). Analysis of the genetic variability of virulence-related loci in epidemic clones of methicillin-resistant *Staphylococcus aureus*. *Antimicrob. Agents Chemother.* 49 366–379. 10.1128/aac.49.1.366-379.2005 15616317 PMC538922

[B11] Holtfreter S. Grumann D. Schmudde M. Nguyen H. Eichler P. Strommenger B. (2007). Clonal distribution of superantigen genes in clinical *Staphylococcus aureus* isolates. *J. Clin. Microbiol.* 45 2669–2680. 10.1128/jcm.00204-07 17537946 PMC1951235

[B12] Jarraud S. Lyon G. Figueiredo A. Lina G. Gérard L. Vandenesch F. (2000). Exfoliatin-producing strains define a fourth agr specificity group in *Staphylococcus aureus*. *J. Bacteriol.* 182 6517–6522. 10.1128/jb.182.22.6517-6522.2000 11053400 PMC94802

[B13] Jarraud S. Mougel C. Thioulouse J. Lina G. Meugnier H. Forey F. (2002). Relationships between *Staphylococcus aureus* genetic background, virulence factors, agr groups (alleles), and human disease. *Infect. Immun.* 70 631–641. 10.1128/iai.70.2.631-641.2002 11796592 PMC127674

[B14] Ji G. Beavis R. Novick R. (1995). Cell density control of staphylococcal virulence mediated by an octapeptide pheromone. *Proc. Natl. Acad. Sci. U.S.A.* 92 12055–12059. 10.1073/pnas.92.26.12055 8618843 PMC40295

[B15] Ji G. Beavis R. Novick R. (1997). Bacterial interference caused by autoinducing peptide variants. *Science* 276 2027–2030. 10.1126/science.276.5321.2027 9197262

[B16] Johansson C. Rautelin H. Kaden R. (2019). *Staphylococcus argenteus* and are cytotoxic to human cells due to high expression of alpha-hemolysin Hla. *Virulence* 10 502–510. 10.1080/21505594.2019.1620062 31131704 PMC6550535

[B17] Khoramrooz S. Mansouri F. Marashifard M. Malek Hosseini S. Akbarian Chenarestane-Olia F. Ganavehei B. (2016). Detection of biofilm related genes, classical enterotoxin genes and agr typing among *Staphylococcus aureus* isolated from bovine with subclinical mastitis in southwest of Iran. *Microbial. Pathog.* 97 45–51. 10.1016/j.micpath.2016.05.022 27251096

[B18] Kullik I. Giachino P. Fuchs T. (1998). Deletion of the alternative sigma factor sigma B in *Staphylococcus aureus* reveals its function as a global regulator of virulence genes. *J. Bacteriol.* 180 4814–4820. 10.1128/JB.00536-07 9733682 PMC107504

[B19] Liu H. Shang W. Hu Z. Zheng Y. Yuan J. Hu Q. (2018). A novel SigB(Q225P) mutation in *Staphylococcus aureus* retains virulence but promotes biofilm formation. *Emerg. Microbes Infect.* 7:72. 10.1038/s41426-018-0078-1 29691368 PMC5915575

[B20] Majerczyk C. Dunman P. Luong T. Lee C. Sadykov M. Somerville G. (2010). Direct targets of CodY in *Staphylococcus aureus*. *J. Bacteriol.* 192 2861–2877. 10.1128/jb.00220-10 20363936 PMC2876493

[B21] Manna A. C. Cheung A. L. (2003). sarU, a sarA homolog, is repressed by SarT and regulates virulence genes in *Staphylococcus aureus*. *Infect. Immun.* 71 343–353. 10.1128/IAI.71.1.343-353.2003 12496184 PMC143423

[B22] Manna A. C. Cheung A. L. (2006). Expression of SarX, a Negative regulator of agr and exoprotein synthesis, Is activated by MgrA in *Staphylococcus aureus*. *J. Bacteriol.* 188 4288–4299. 10.1128/JB.00297-06 16740935 PMC1482969

[B23] Mei G. L. Cue D. Roux C. M. Dunman P. M. Lee C. Y. (2011). Rsp inhibits attachment and biofilm formation by repressing fnbA in *Staphylococcus aureus* MW2. *J. Bacteriol.* 193 5231–5241. 10.1128/JB.05454-11 21804010 PMC3187379

[B24] Novick R. Projan S. Kornblum J. Ross H. Ji G. Kreiswirth B. (1995). The agr P2 operon: an autocatalytic sensory transduction system in *Staphylococcus aureus*. *Mol. Gen. Genet.* 248 446–458. 10.1007/bf02191645 7565609

[B25] Pader V. James E. Painter K. Wigneshweraraj S. Edwards A. (2014). The Agr quorum-sensing system regulates fibronectin binding but not hemolysis in the absence of a functional electron transport chain. *Infect. Immun*. 82 4337–4347. 10.1128/IAI.02254-14 25092909 PMC4187888

[B26] Pelz A. Wieland K. Putzbach K. Hentschel P. Albert K. Götz F. (2005). Structure and biosynthesis of staphyloxanthin from *Staphylococcus aureus*. *J. Biol. Chem.* 280 32493–32498. 10.1074/jbc.M505070200 16020541

[B27] Powers M. Becker R. Sailer A. Turner J. Bubeck Wardenburg J. (2015). Synergistic action of *Staphylococcus aureus* α-Toxin on platelets and myeloid lineage cells contributes to lethal sepsis. *Cell Host Microbe* 17 775–787. 10.1016/j.chom.2015.05.011 26067604 PMC4642999

[B28] Queck S. Jameson-Lee M. Villaruz A. Bach T. Khan B. Sturdevant D. (2008). RNAIII-independent target gene control by the agr quorum-sensing system: insight into the evolution of virulence regulation in *Staphylococcus aureus*. *Mol. Cell* 32 150–158. 10.1016/j.molcel.2008.08.005 18851841 PMC2575650

[B29] Rao Q. Zhou K. Zhang X. Hu Q. Zhu J. Chen Z. (2015). Fatal multiple organ failure in an adolescent due to community-acquired methicillin-susceptible *Staphylococcus aureus* ST121/agrIV lineage: a case report. *Rev. Med. Microbiol.* 26:1. 10.1097/MRM.0000000000000050

[B30] Reyes-Robles T. Torres V. (2017). Staphylococcus aureus pore-forming toxins. *Curr. Topics Microbiol. Immunol.* 409 121–144. 10.1007/82_2016_16 27406190

[B31] Seilie E. Bubeck Wardenburg J. (2017). Staphylococcus aureus pore-forming toxins: the interface of pathogen and host complexity. *Semin. Cell Dev. Biol.* 72 101–116. 10.1016/j.semcdb.2017.04.003 28445785 PMC5823538

[B32] Singh R. Ray P. (2014). Quorum sensing-mediated regulation of staphylococcal virulence and antibiotic resistance. *Future Microbiol.* 9 669–681. 10.2217/fmb.14.31 24957093

[B33] Tan L. Li S. Jiang B. Hu X. Li S. (2018). Staphylococcus aureusTherapeutic Targeting of the Accessory Gene Regulator (agr) System. *Front. Microbiol.* 9:55. 10.3389/fmicb.2018.00055 29422887 PMC5789755

[B34] Traber K. Lee E. Benson S. Corrigan R. Novick R. (2008). Agr function in clinical *Staphylococcus aureus* isolates. *Microbiology* 154 2265–2274. 10.1099/mic.0.2007/011874-0 18667559 PMC4904715

[B35] Tsompanidou E. Sibbald M. Chlebowicz M. Dreisbach A. Back J. van Dijl J. (2011). Requirement of the agr locus for colony spreading of *Staphylococcus aureus*. *J. Bacteriol.* 193 1267–1272. 10.1128/JB.01276-10 21169484 PMC3067592

[B36] Vandenesch F. Lina G. Henry T. (2012). *Staphylococcus aureus* hemolysins, bi-component leukocidins, and cytolytic peptides: a redundant arsenal of membrane-damaging virulence factors? *Front. Cell. Infect. Microbiol.* 2:12. 10.3389/fcimb.2012.00012 22919604 PMC3417661

[B37] Wang B. Muir T. (2016). Regulation of virulence in *Staphylococcus aureus*: molecular mechanisms and remaining puzzles. *Cell Chem. Biol.* 23 214–224. 10.1016/j.chembiol.2016.01.004 26971873 PMC4847544

[B38] Wieland B. Feil C. Gloria-Maercker E. Thumm G. Lechner M. Bravo J. (1994). Genetic and biochemical analyses of the biosynthesis of the yellow carotenoid 4,4’-diaponeurosporene of *Staphylococcus aureus*. *J. Bacteriol.* 176 7719–7726. 10.1128/jb.176.24.7719-7726.1994 8002598 PMC197231

[B39] Wright J. Traber K. Corrigan R. Benson S. Musser J. Novick R. (2005). The agr radiation: an early event in the evolution of staphylococci. *J. Bacteriol.* 187 5585–5594. 10.1128/jb.187.16.5585-5594.2005 16077103 PMC1196086

[B40] Xue L. Chen Y. Yan Z. Lu W. Zhu H. (2019). Staphyloxanthin: a potential target for antivirulence therapy. *Infect. Drug Resist.* 12 2151–2160. 10.2147/IDR.S193649 31410034 PMC6647007

[B41] Yuan W. Hu Q. Cheng H. Shang W. Liu N. Hua Z. (2013). Cell wall thickening is associated with adaptive resistance to amikacin in methicillin-resistant *Staphylococcus aureus* clinical isolates. *J. Antimicrob. Chemother.* 68 1089–1096. 10.1093/jac/dks522 23322605

