# Supplementary figures and images for: Accessory Gene Regulator (agr) Allelic Variants in Cognate Staphylococcus aureus Strain Display Similar Phenotypes
Source: Front Microbiol. 2022 Feb 25;13:700894. doi: 10.3389/fmicb.2022.700894 (PMC8919982; doi:10.3389/fmicb.2022.700894)

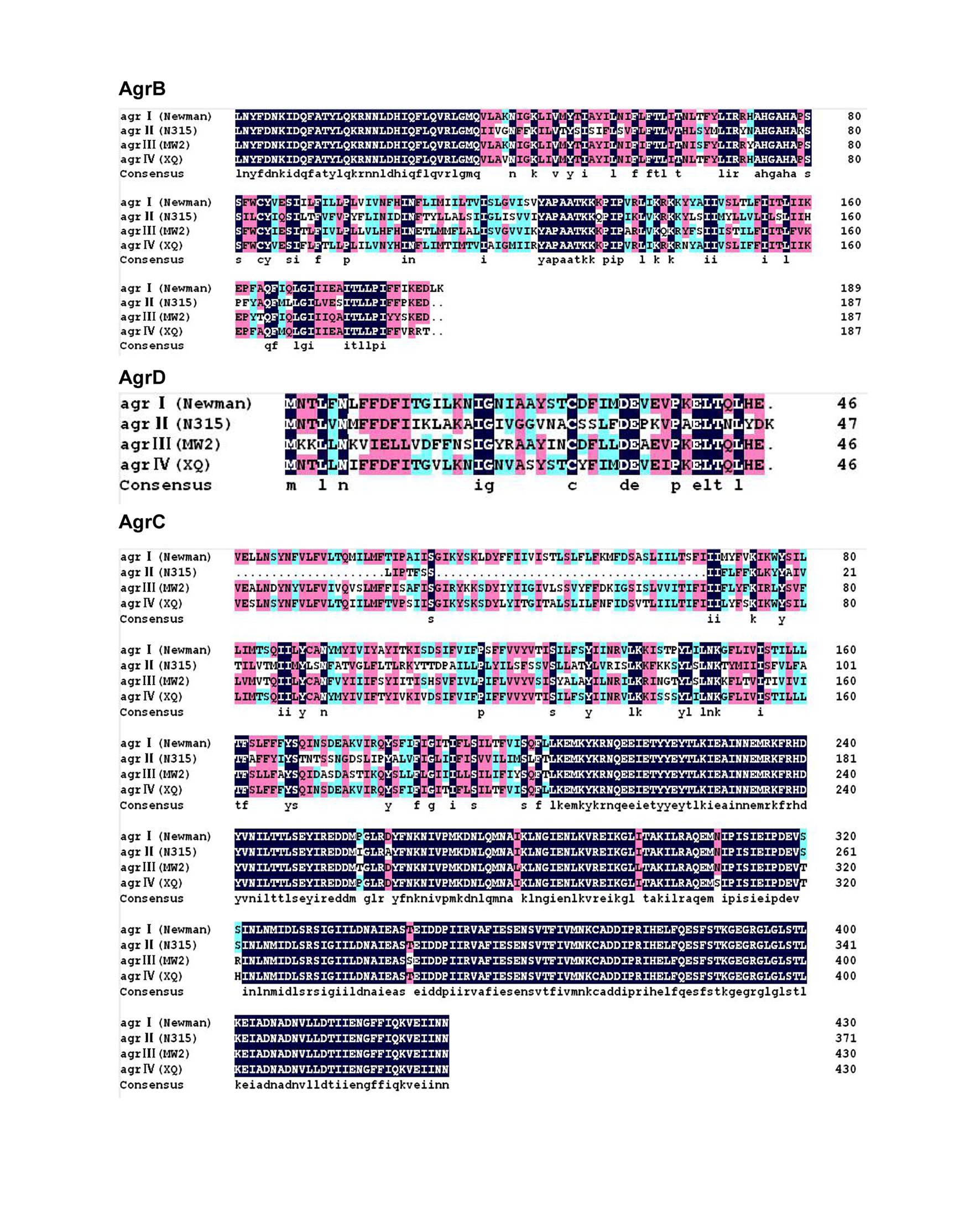

Supplement: Supplementary Figure 1 — The amino acids comparisons of AgrB (A), AgrD (B), and AgrC (C) among the four S. aureus agr allelic strains: agrI (Newman), agrII (N315), agrIII (MW2), and agrIV (XQ). The sequences were obtained from NCBI (National Center for Biotechnology Information) database and aligned with the Clustal X and DNAMAN software. The blast results show that the amino acid sequences of AgrB, AgrD, and AgrC are variable. [file Image_1.TIF]

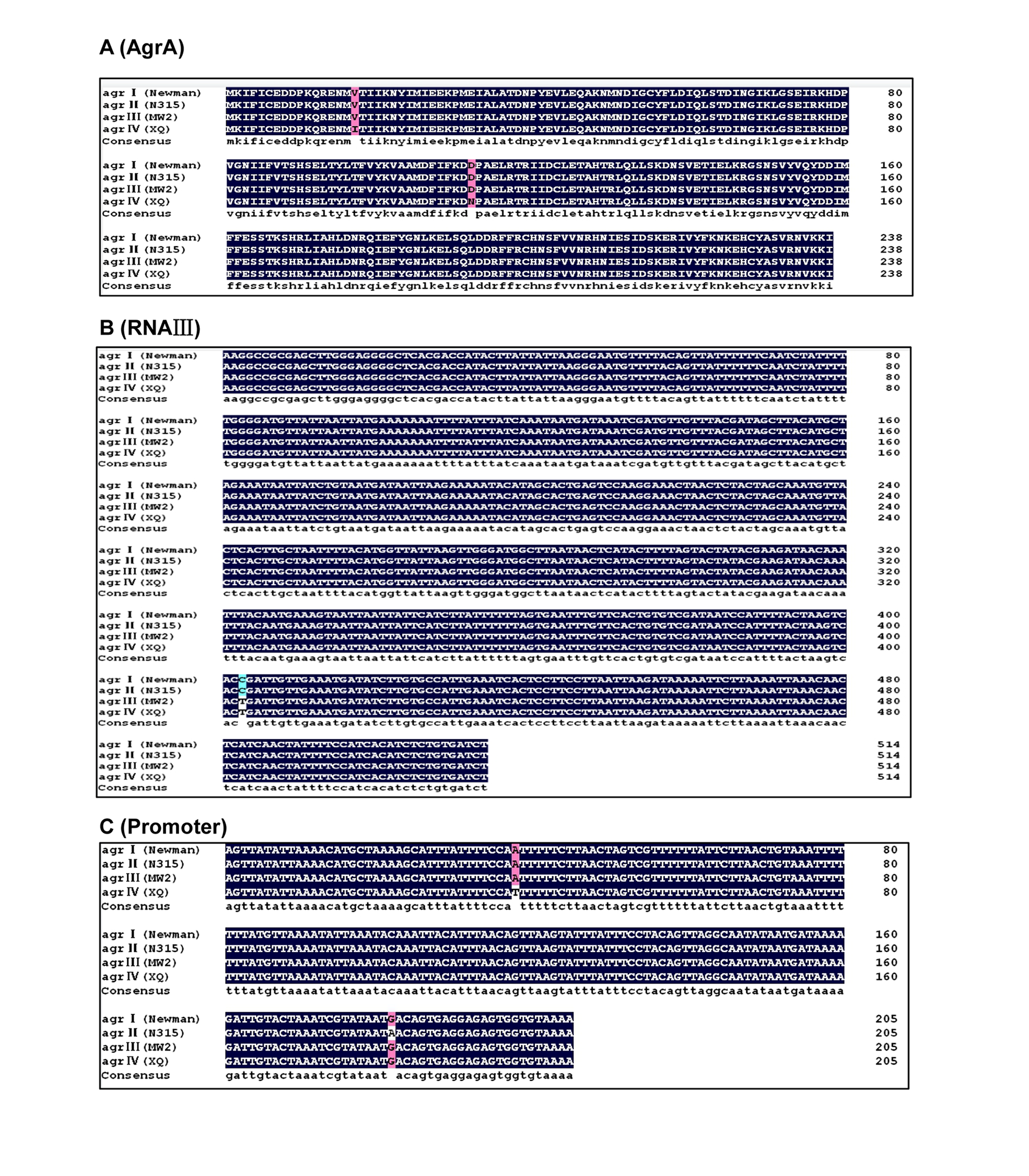

Supplement: Supplementary Figure 2 — The comparisons of AgrA (A), RNAIII (B), and promoter sequences (C) among the four S. aureus agr allelic strains: agrI (Newman), agrII (N315), agrIII (MW2), and agrIV (XQ). The sequences were obtained from NCBI (National Center for Biotechnology Information) database (http://www.ncbi.nlm.nih.gov) and aligned with the Clustal X and DNAMAN software. The blast results show that the AgrA, RNAIII, and promoter region are highly conserved among the four S. aureus agr allelic strains. [file Image_2.TIF]

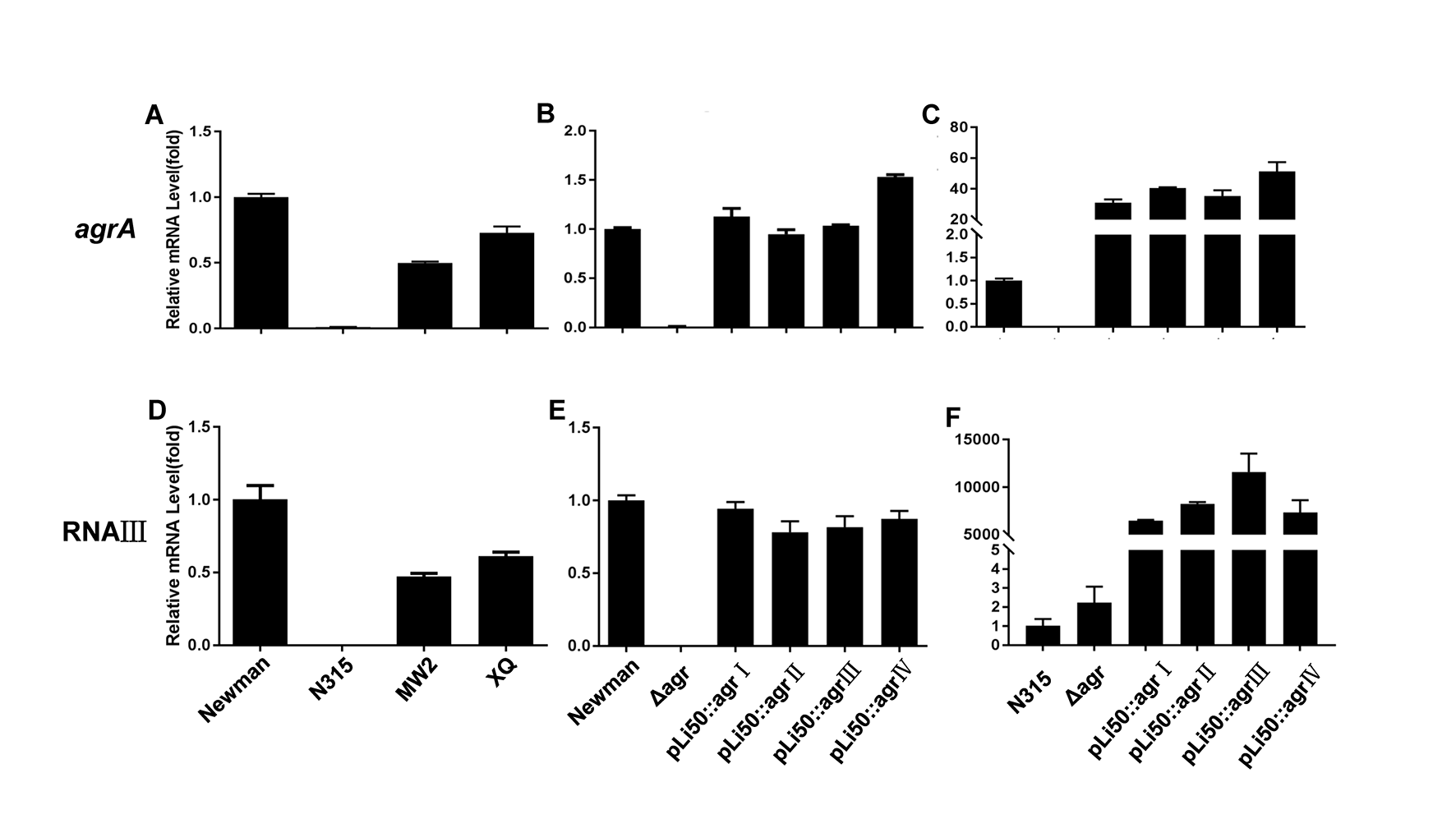

Supplement: Supplementary Figure 3 — Effects of agr alleles on agr transcriptions. The total bacterial RNA was isolated in mid-log phase, and gene transcription was appraised with quantitative RT-PCR. The transcription of agrA (A–C) and RNAIII (D–F) among the four wild agr allele strains (agrI/Newman, agrII/N315, agrIII/MW2, and agrIV/XQ) (left) and agr plasmid complemented congenic strains in Newman (middle) or N315 (right) background were analyzed. Error bars represent the average standard deviation (SD) of three separate experiments. [file Image_3.TIF]

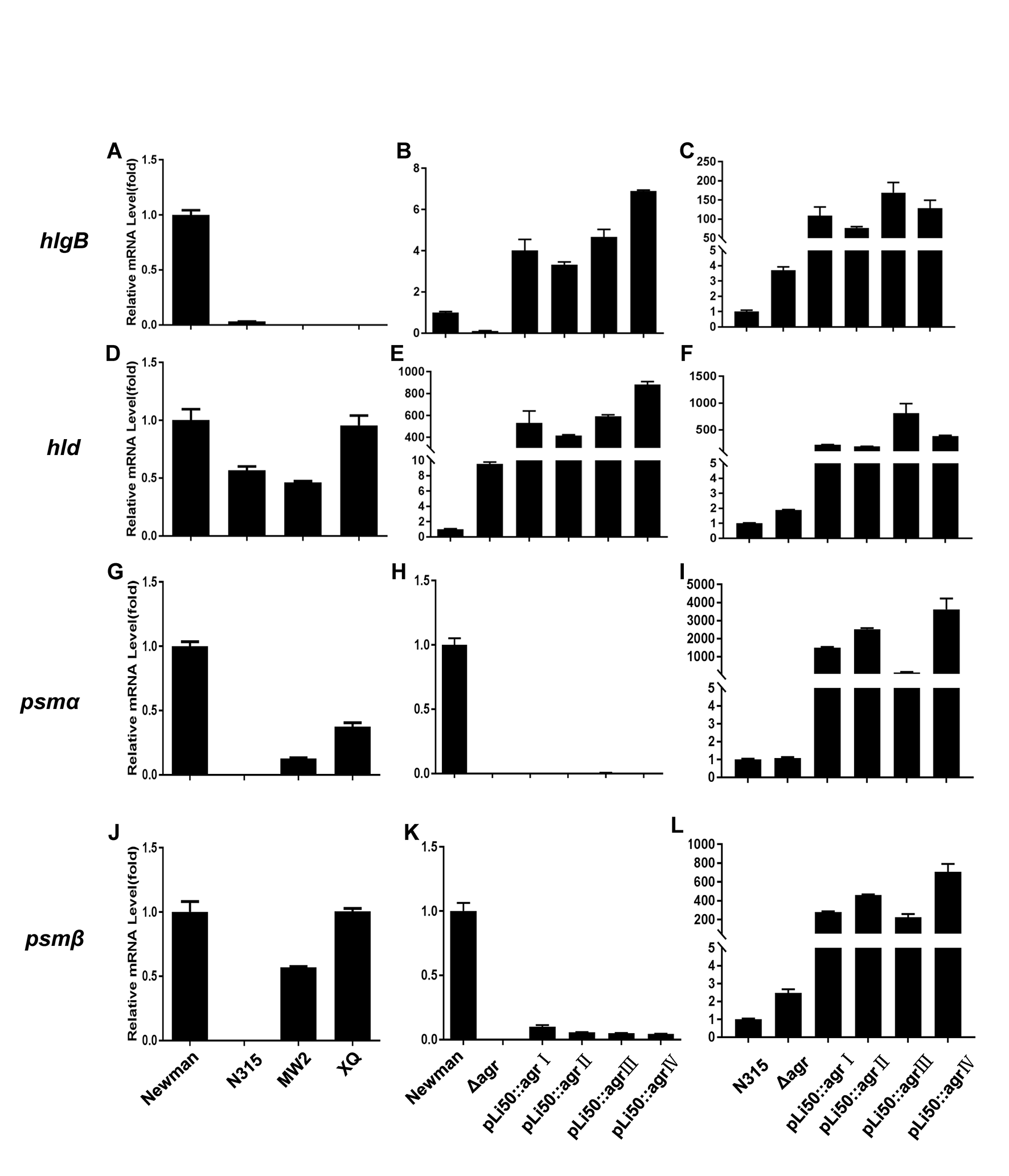

Supplement: Supplementary Figure 4 — Effects of agr alleles on virulence gene transcriptions of S. aureus. The total bacterial RNA was isolated in mid-log phase, and gene transcription was appraised with quantitative RT-PCR. The transcription of hlgB (A–C), hld (D–F), psmα (G–I), and psmβ (J–L) among the four wild agr allelic strains (agrI/Newman, agrII/N315, agrIII/MW2, and agrIV/XQ) (left); agr plasmid complemented congenic strains in Newman (middle); or N315 (right) background were analyzed. Error bars represent the average standard deviation (SD) of three separate experiments. [file Image_4.TIF]
